# Association between diet quality and the oral microbiome in three US cohort studies

**DOI:** 10.1080/20002297.2026.2635238

**Published:** 2026-02-24

**Authors:** Fangyu Li, Samuel Anyaso-Samuel, Yukiko Yano, Vicky C. Chang, Xing Hua, Yunhu Wan, Casey L. Dagnall, Kristine Jones, Belynda D. Hicks, Amy Hutchinson, Linda M. Liao, Wen-Yi Huang, Neal D. Freedman, Laura E. Beane Freeman, Dale P. Sandler, Christian C. Abnet, Rashmi Sinha, Jianxin Shi, Erikka Loftfield, Emily Vogtmann

**Affiliations:** a Division of Cancer Epidemiology & Genetics, National Cancer Institute, National Institutes of Health, Bethesda, MD, USA; b Frederick National Laboratory for Cancer Research/Leidos Biomedical Research Laboratory, Inc., Frederick, MD, USA; c Division of Cancer Control & Population Science, National Cancer Institute, National Institutes of Health, Bethesda, MD, USA; d Epidemiology Branch, Chronic Disease Epidemiology Group, National Institute for Environmental Health Science, National Institutes of Health, Research Triangle Park, NC, USA

**Keywords:** Diet quality, healthy eating index, oral microbiome, cohort study, cancer risk

## Abstract

**Background:**

The oral microbiome has been associated with overall health, but the contribution of dietary habits to oral microbial composition is not well understood.

**Objective:**

We evaluated the association between diet quality (Healthy Eating Index [HEI] 2015) and the oral microbiome in the Agricultural Health Study, NIH-AARP Diet and Health Study, and Prostate, Lung, Colorectal, and Ovarian Cancer Screening Trial within 5,546 participants.

**Methods:**

Individual HEI components were scored from FFQ data and summed. Alpha and beta diversity and genus-level presence and relative abundance were estimated. The proportion of variability in the beta diversity matrices explained by diet quality and other covariates were calculated. Linear, logistic, and zero-inflated negative binomial regression models with adjustment for confounders were used and cohort-specific estimates were meta-analyzed.

**Results:**

Age explained the largest variability in beta diversity (Bray-Curtis), followed by smoking, education, and the HEI component for added sugar. Although overall diet quality was not associated with alpha diversity overall, the added sugar component was consistently inversely associated with alpha diversity. At the genus-level, most of the identified associations were with added sugar.

**Conclusions:**

Consumption of added sugars was consistently associated with oral microbial diversity and specific genera.

## Introduction

The oral microbiome, a diverse and dynamic community of bacteria, fungi, viruses, and other microorganisms inhabiting the oral cavity, likely plays a fundamental role in maintaining oral and systemic health [1]. This microbial ecosystem contributes to essential functions such as digestion, immune modulation, and protection against pathogenic colonization [2]. However, specific bacteria or an overall imbalance in the composition of the oral microbiome has been implicated in the pathogenesis of various diseases, including periodontal disease, cardiovascular conditions, diabetes, and notably, several types of cancer [1]. Emerging evidence has linked aspects of the oral microbiome to greater risks of colorectal [3], lung [4], and head and neck cancers [5], suggesting that microbial communities in the mouth may influence carcinogenesis through mechanisms such as inflammation, direct interaction with the tissue, and production of carcinogenic metabolites. Despite these advances, the factors that shape the composition and diversity of the oral microbiome remain incompletely understood, with genetics, environmental exposures, and lifestyle factors all potentially playing contributory roles [2].

Among lifestyle factors, diet may be a critical and modifiable determinant of microbial ecology in the oral cavity [2]. Marked shifts in the oral microbiome have been detected in dental calculus from ancient human skeletons and are hypothesized to reflect the evolution of diet from subsistence hunter-gatherer culture to industrial farming [6]. While many studies have investigated how the gut microbiome responds to diet [7–9], the impact of diet on the oral microbiome has received comparatively less attention. The Healthy Eating Index (HEI) is a measure of diet quality used to assess adherence to the Dietary Guidelines for Americans by capturing the balance of adequacy components, which represent dietary factors that are encouraged, and moderation components, which represent dietary factors with recommended intake limits [10]. Higher diet quality has been consistently associated with favorable health outcomes, including lower risk of cardiovascular disease, and cancer, and mortality [11–13]. Diet quality has been found to be associated with oral diseases, but these associations are likely bi-directional [14–16]. The extent to which diet quality shapes the oral microbiome, and which specific dietary components exert the strongest influence, remains to be fully elucidated. Understanding of these relationships is crucial for identifying potential dietary interventions that could modulate the oral microbiome to promote health and reduce disease risk. Although interventional studies will provide the most compelling evidence, with the limited knowledge and many dietary components to consider, large observational studies can efficiently improve our current understanding.

The objective of our study was to evaluate the association of diet quality, measured using the HEI, and its components with the oral microbiome using data from oral microbiome studies nested within three prospective US cohort studies that employed the same dietary assessment questionnaire: the Agricultural Health Study (AHS) [17], the NIH-AARP Diet and Health Study [18], and the Prostate, Lung, Colorectal, and Ovarian (PLCO) Cancer Screening Trial [19].

## Methods

### Study population

We conducted a nested case-cohort study of the oral microbiome and risk of eight cancers within the AHS (NCT00352924), NIH-AARP (NCT04750915), and PLCO (NCT00339495) studies with detailed descriptions published previously [3,4]. In brief, the AHS is a prospective cohort study of 89,655 licensed private or commercial pesticide applicators and their spouses recruited in Iowa and North Carolina from 1993 to 1997 [17]. The NIH-AARP is a prospective cohort study of 566,398 AARP members residing in the states of California, Florida, Louisiana, New Jersey, North Carolina, and Pennsylvania, or in the cities of Atlanta, Georgia and Detroit, Michigan with enrollment from 1995 to 1996 [18]. The PLCO is a randomized screening trial of over 150,000 individuals recruited from ten screening centers in the US from 1993 to 2001 [19]. From the three cohorts, demographic and lifestyle data were collected using standardized, cohort-specific questionnaires administered at baseline or during follow-up visits. Oral wash specimens were collected from a subset of participants from the AHS starting in 1999, NIH-AARP in 2005, and the PLCO control arm from 2000−2003. We identified individuals in these cohorts with an oral wash specimen who developed incident cancer of the lung, colorectum, esophagus, head/neck, stomach, small intestine, pancreas, or hepatobiliary tract through 2013, 2011, or 2009 for AHS, NIH-AARP, and PLCO, respectively. We then selected a comparison referent subcohort based on stratum-specific sampling weights to ensure sufficient individuals for comparison based on age, sex, and smoking status. Incident cancer cases were assigned a weight of 1 and individuals in the referent subcohort were assigned a sampling weight based on the inverse of the observed sampling fractions.

For this analysis, we included participants with available oral microbiome data who completed a valid food frequency questionnaire (FFQ). After excluding participants with missing demographic, lifestyle, dietary, or microbiome data, prevalent cancer at oral wash collection, and those with extreme caloric intake (< 600 kcal or > 5000 kcal), our final analytic sample included 1,452 participants from the AHS cohort (weighted *N* = 26,469), 1,921 participants from the NIH-AARP cohort (weighted *N* = 24,958), and 2,173 participants (weighted *N* = 34,842) from the PLCO cohort (**Supplemental Figure 1**). Individuals excluded due to missing lifestyle or dietary information and those with self-reported extreme caloric intake in AHS and PLCO were more likely to be male, have less education, report a race and ethnicity other than non-Hispanic White, and be a current smoker. No differences were observed for excluded participants in the NIH-AARP cohort. Each cohort study was approved by the Special Studies Institutional Review Board at the National Cancer Institute, and PLCO was also approved by the Institutional Review Board at each of the 10 screening centers. Informed consent was obtained from participants or implied from completion and return of study questionnaires.

### Diet assessment

Dietary intake was assessed in each cohort using a validated FFQ designed for US populations [20–22]. Usual dietary intake in the past year was assessed using the Dietary History Questionnaire (DHQ), which consists of 124 food items and includes questions on portion size, in AHS from 1999−2003 and in PLCO from 1998−2000. The FFQ used in the NIH-AARP was an early version of the 124-item DHQ and was included in the baseline questionnaire from 1995−1996 [22]. The MyPyramid Equivalents Database was used to derive food group equivalents and generate nutrient and energy intake estimates using the US Department of Agriculture (USDA) Survey Nutrient Database, which was based on national dietary data from the 1994−1996 USDA Continuing Survey of Food Intakes of Individuals survey [23].

Diet quality was quantified using the HEI-2015, a validated measure that assesses adherence to the 2015−2020 Dietary Guidelines for Americans [10,24]. The HEI-2015 includes 13 components that capture adequacy (i.e. total fruits, whole fruits, total vegetables, greens and beans, whole grains, dairy, total protein foods, seafood and plant proteins, and fatty acids) and moderation (i.e. refined grains, sodium, added sugars, and saturated fats) components. Most of the components are scored on a density basis (per 1,000 kcal or as a percentage of energy intake) where a higher score for adequacy components indicates greater consumption while a higher score for moderation components indicates lower consumption. Each component score is summed to yield a total score ranging from 0 to 100 with higher scores indicating better diet quality. Individual HEI component scores were included in analyses to explore specific dietary associations with the oral microbiome. Given the observed associations with the HEI component score for added sugars, we additionally investigated the associations with percent energy from added sugars for more straightforward interpretation.

### Laboratory handling and bioinformatics

The procedures for DNA extraction, PCR amplification, sequencing, and bioinformatics have been previously described in detail [4]. In summary, DNA was extracted from the buccal cell pellets using the DSP DNA Virus Pathogen kit (Qiagen). PCR amplification was conducted using barcoded primers targeting the 16S rRNA gene V4 region. Sequencing of the DNA was performed using the MiSeq platform (Illumina) with 2 × 250-bp paired-end reads. The sequencing data from the three cohorts were processed independently using DADA2 using QIIME 2 version 2018.4 [25] to generate amplicon sequence variants (ASVs) [26] and taxonomy was assigned using the SILVA v132 database [27]. Alpha diversity metrics (i.e. within sample diversity), including observed ASVs, the Shannon index, and Faith's phylogenetic diversity (PD), and beta diversity metrics (i.e. between sample microbial community dissimilarity), including Bray-Curtis dissimilarity, and unweighted and weighted UniFrac distances, were calculated based on even sampling at 20,000 reads per sample. Genus-level presence and relative abundances were also estimated.

### Statistical analysis

Unweighted counts with weighted proportions and weighted means and standard deviations (SD) were calculated for the demographic (i.e. age at oral sample collection, sex, education level, self-identified race and ethnicity) and lifestyle (i.e. body mass index [BMI] and smoking status) factors, HEI-2015, HEI-2015 components, other dietary factors (i.e. consumption of coffee, tea, or alcohol, and total energy intake), and alpha diversity metrics for each cohort. We quantified the proportion of variability in each of the beta diversity matrices attributable to HEI-2015, its components, and selected demographic and lifestyle factors using the FastAdonis function, which performs a weighted permutational multivariate analysis of variance (PERMANOVA) while accounting for the complex sampling design [28].

Associations between total and individual HEI-2015 component scores and three alpha diversity metrics (i.e. observed ASVs, Faith's PD, and the Shannon Index) were calculated using stratified, weighted, multivariable linear regression models with adjustment for all potential confounders for each cohort separately (i.e. dietary and lifestyle factors). Results from each cohort were combined using fixed-effect meta-analysis to estimate the pooled beta coefficients (*β*), 95% confidence intervals (CI), and nominal *p*-values. Between-study heterogeneity was assessed using Cochran's Q test.

For the genus-level associations, we employed stratified, weighted zero-inflated negative binomial regression models to jointly model the presence and relative abundance of each genus by cohort with adjustment for all potential confounders. This analysis was restricted to genera that were present in 5−95% of the populations with a weighted relative abundance greater than 0.1% (*N* = 21 genera). The genus-level sequence counts were included as the outcome with the total sequence counts as the offset. Given that the zero-inflated portion of the model estimates the association with the absence of the genus (i.e. the opposite of logistic regression models), we took the inverse of the odds ratio (OR) from the output to model the associations with the presence of the genus. The negative binomial output of the model is presented as ratios of genus-level abundance (RR) among those with presence of the genus. For genera with a prevalence ranging from 5−95% but with a weighted relative abundance less than or equal to 0.1% (*N* = 60 genera), we used stratified, weighted binary logistic regression models to estimate ORs for the associations between the HEI-2015 components and the presence of each genus with adjustment for confounders. Finally, for genera with a prevalence greater than 95% (*N* = 20 genera), we applied stratified, weighted linear regression models of the centered log-ratio (CLR)-transformed relative abundance data with adjustment for confounders to account for compositionality of relative abundance data. All cohort-specific estimates were combined using fixed-effects meta-analysis. To control for multiple testing across all genera (*N* = 101), we applied false-discovery rate (FDR) correction to the *p*-values using the Benjamini Hochberg procedure, and an FDR *p*-value less than 0.05 considered statistically significant. All statistical analyses were conducted using R software (version 4.3.1), with the exception of the zero-inflated negative binomial models which were conducted using Stata (version 18.0), and all models incorporated the complex sampling design.

## Results

### Population characteristics

Participant characteristics varied across the three cohorts (Table 1). Participants in the AHS were on average the youngest at sample collection (54.4, SD 11.5 years), while the participants in NIH-AARP were oldest (71.0, SD 5.3 years). The proportion of females was highest in PLCO (54.0%), followed by AHS (45.8%) and NIH-AARP (40.2%). The NIH-AARP cohort had the highest proportion of individuals with BMI < 25 kg/m^2^ (38.0%) and the greatest proportion of current or former smokers (58.7%). For diet quality, HEI-2015 scores were the highest on average among participants in the NIH-AARP (68.8, SD 9.4) and lowest among participants in the AHS (61.4, SD 9.6). The highest average alpha diversity estimates were all observed among NIH-AARP participants. In contrast, PLCO participants had the lowest diversity estimates for observed ASVs (122.9, SD 38.6) and Faith's PD (9.21, SD 1.93), while participants in AHS had the lowest diversity measured by the Shannon Index (4.36, SD 0.74).

**Table 1.** Demographic, lifestyle, dietary, and oral microbiome characteristics of the participants in the Agricultural Health Study (AHS), NIH-AARP Diet and Health Study, and Prostate, Lung, Colorectal, and Ovarian Cancer Screening Trial (PLCO) with oral microbiome data.

	AHS	NIH-AARP	PLCO
	Unweighted *N* = 1,452	Unweighted *N* = 1,921	Unweighted *N* = 2,173
	Weighted *N* = 26,469	Weighted *N* = 24,958	Weighted *N* = 34,842
**Age at sample collection, years, mean (SD)**	54.4 (11.5)	71.0 (5.3)	67.0 (5.7)
**Sex, *n* (%)**			
Female	561 (45.8%)	720 (40.2%)	1,068 (54.0%)
Male	891 (54.3%)	1,201 (59.8%)	1,105 (46.0%)
**BMI, kg/m2, *n* (%)**			
<25	344 (26.3%)	723 (38.0%)	732 (33.0%)
25.0 to <30.0	428 (29.5%)	861 (44.1%)	968 (45.0%)
> = 30	217 (12.9%)	337 (17.9%)	473 (22.1%)
Missing	463 (31.3%)	0 (0.0%)	0 (0.0%)
**Education, *n* (%)**			
<High school	113 (6.3%)	91 (3.9%)	154 (4.8%)
High school	661 (41.0%)	314 (15.9%)	516 (21.3%)
Some college	341 (26.0%)	655 (33.0%)	772 (34.7%)
College graduate	258 (21.1%)	861 (47.2%)	731 (39.2%)
Missing	79 (5.6%)	0 (0.0%)	0 (0.0%)
**Race and ethnicity, *n* (%)**			
Non-Hispanic White	1,420 (98.0%)	1,810 (93.9%)	2,048 (94.3%)
Other races and ethnicities	32 (2.0%)	111 (6.1%)	125 (5.7%)
**Smoking status, *n* (%)**			
Current smoker	327 (9.7%)	458 (9.6%)	606 (9.1%)
Former smoker	503 (26.0%)	1,029 (49.1%)	924 (42.9%)
Never smoker	540 (57.4%)	350 (35.9%)	581 (43.3%)
Never smoker but used cigars	48 (4.8%)	31 (2.7%)	62 (4.7%)
Missing	34 (2.1%)	53 (2.7%)	0 (0.0%)
**Dietary factors, mean (SD)**			
Coffee, cups/day	1.43 (1.68)	2.08 (1.70)	1.96 (1.83)
Tea, cups/day	0.16 (0.48)	0.48 (0.98)	0.35 (0.79)
Alcoholic drinks, drinks/day	0.30 (0.76)	0.92 (2.19)	0.68 (1.89)
Total energy, kcal/day	1,968 (801)	1,811 (717)	1,724 (716)
% energy from added sugar	13.1 (6.2)	9.3 (6.0)	10.9 (5.5)
**Total HEI-2015, mean (SD)**	61.4 (9.6)	68.8 (9.4)	67.3 (9.6)
**HEI-2015 adequacy components, mean (SD)**			
Total fruit	3.80 (1.46)	4.26 (1.29)	4.26 (1.24)
Whole fruit	4.45 (1.14)	4.28 (1.30)	4.41 (1.17)
Total vegetables	4.14 (1.01)	4.15 (1.06)	4.35 (0.94)
Greens and beans	1.79 (1.36)	3.24 (1.54)	2.63 (1.59)
Whole grains	3.17 (2.16)	3.93 (2.50)	3.94 (2.41)
Total dairy	5.98 (3.03)	5.48 (2.89)	5.71 (2.97)
Total protein foods	4.55 (0.77)	4.43 (0.88)	4.42 (0.85)
Seafood and plant proteins	3.33 (1.35)	3.97 (1.19)	3.91 (1.24)
Fatty acids (UFAs/SFAs)	4.74 (2.56)	6.08 (2.84)	5.67 (2.73)
**HEI-2015 moderation components, mean (SD)**			
Refined grains	8.09 (2.24)	7.48 (2.62)	8.43 (1.99)
Sodium	4.62 (2.45)	5.50 (2.59)	4.73 (2.59)
Added sugar	6.70 (2.70)	8.24 (2.48)	7.63 (2.39)
Saturated fats	6.04 (2.86)	7.77 (2.67)	7.22 (2.78)
**Alpha diversity, mean (SD)**			
Observed ASVs	124.9 (44.5)	128.8 (42.0)	122.9 (38.6)
Faith's PD	10.35 (2.46)	10.56 (2.36)	9.21 (1.93)
Shannon Index	4.36 (0.74)	4.58 (0.65)	4.46 (0.63)

Note: Table counts are unweighted, but all percentages, means, and standard deviations (SD) are weighted estimates.

Abbreviations: Amplicon sequence variants (ASVs), phylogenetic diversity (PD), saturated fatty acids (SFAs), unsaturated fatty acids (UFAs).

### Variability explained in beta diversity by demographic, lifestyle, and dietary factors

**Figure 1.**
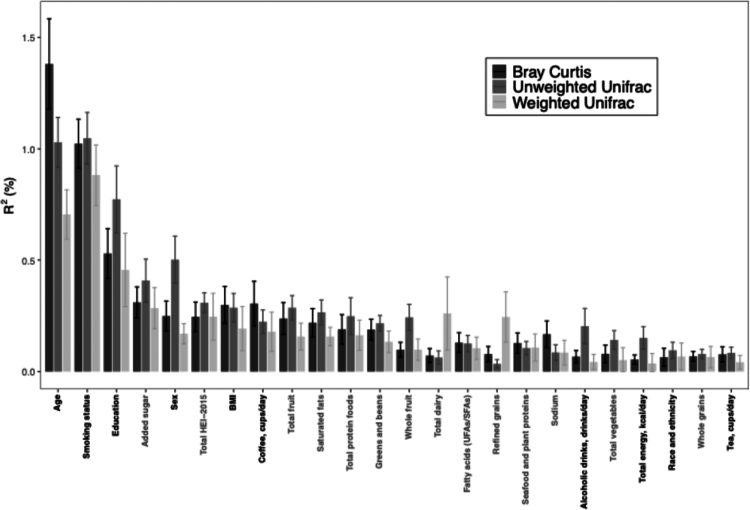
Variability in the Bray-Curtis and unweighted and weighted UniFrac beta diversity matrices explained by demographic, lifestyle, dietary, and Healthy Eating Index (HEI) variables. For the HEI variables, the total HEI-2015 score has a maximum of 100 points, whole grains, dairy, fatty acids (UFAs/SFAs), refined grains, sodium, added sugars, and saturated fats have a maximum of 10 points, and total fruits, whole fruits, total vegetables, greens and beans, total protein foods, and seafood and plant proteins have a maximum of 5 points. Variability explained by these variables was calculated using the FastAdonis function. In total, 4.66%, 4.93%, and 3.92% of the variability in Bray-Curtis, unweighted UniFrac, and weighted UniFrac, respectively, were explained by these factors.

As illustrated in Figure 1, among all of the demographic, lifestyle, dietary, and HEI variables, age explained the largest proportion of variability in the Bray-Curtis distance matrix (1.38%), followed by smoking status (1.02%), education level (0.53%), and the 10-point HEI component score for added sugar (0.31%) for the three cohorts combined. For unweighted UniFrac, smoking status explained the most variability (1.05%), followed closely by age (1.03%), with the HEI component for added sugar as the fifth most important predictor of variability (0.41%) and the strongest dietary factor. Similar trends were observed for weighted UniFrac (Figure 1). However, only a small proportion of variability in beta diversity was explained by these measured factors (e.g. 4.7% of the Bray-Curtis dissimilarity matrix; **Supplemental Figure 2**).

### HEI-2015 associations with alpha diversity

As shown in Table 2, a one-point increase in total HEI-2015 score was associated with minimally lower alpha diversity, estimated as observed ASVs (β_Meta-analysis_: −0.24; 95% CI: −1.93, 1.45), but the association was not statistically significant (*p* = 0.78). The associations between HEI-2015 score with observed ASVs were essentially null in each of the three cohorts and there was no statistical heterogeneity in the meta-analysis estimate (*p* = 0.34). For the HEI components, few associations with alpha diversity were observed except for associations with total protein foods, added sugar, and saturated fats. The added sugar association had the largest observed magnitude where for each unit increase in the HEI component score for added sugars (representing a decrease in intake of added sugars), the number of observed ASVs increased by 3.94 (95% CI: 2.18, 5.69). Strong associations between the HEI score for added sugar and observed ASVs were also observed in each cohort, with estimates of 2.79 (95% CI: −0.53, 6.12) in AHS, 5.15 (95% CI: 2.05, 8.25) ASVs in the NIH-AARP, and 3.76 (95% CI: 0.98, 6.53) in PLCO. When added sugar was modeled as the percent of energy consumed from added sugar, a similar association was observed, where an increase of 5% of energy from added sugar was associated with 3.10 fewer ASVs (95% CI: −4.53, −1.68). Similar associations were generally observed with the other measures of alpha diversity, Faith's PD and the Shannon Index (**Supplemental Table 1**).

**Table 2.** Linear regression associations between the Healthy Eating Index (HEI) 2015 and the HEI components with observed sequence variants (ASV) as a measure of alpha diversity in the three cohorts.

	Meta-analysis estimate			AHS		NIH-AARP		PLCO	
	Beta (95% CI)	*p*-value	*p*-hetero	Beta (95% CI)	*p*-value	Beta (95% CI)	*p*-value	Beta (95% CI)	*p*-value
**Total HEI-2015**	*−*0.24 (*−*1.93, 1.45)	0.78	0.34	1.33 (−1.96, 4.62)	0.43	0.18 (−2.63, 2.98)	0.90	−1.77 (−4.55, 1.00)	0.21
**HEI−2015 adequacy components**									
Total fruit	*−*0.87 (*−*2.64, 0.91)	0.34	0.18	0.13 (−3.36, 3.62)	0.94	0.53 (−2.31, 3.36)	0.71	−3.14 (−6.13, −0.15)	0.04
Whole fruit	−1.71 (−3.53, 0.11)	0.07	0.24	−0.55 (−3.96, 2.86)	0.75	−0.50 (−3.59, 2.58)	0.75	−3.78 (−6.81, −0.76)	0.01
Total vegetables	1.73 (−0.10, 3.55)	0.06	0.53	2.30 (−1.16, 5.76)	0.19	2.76 (−0.40, 5.92)	0.09	0.42 (−2.52, 3.36)	0.78
Greens and beans	−0.60 (−2.27, 1.06)	0.48	0.46	−1.01 (−4.34, 2.32)	0.55	0.93 (−2.03, 3.90)	0.54	−1.48 (−3.99, 1.03)	0.25
Whole grains	−0.30 (−1.99, 1.39)	0.73	0.07	−3.31 (−6.86, 0.24)	0.07	1.95 (−0.82, 4.72)	0.17	−0.69 (−3.36, 1.97)	0.61
Total dairy	0.38 (−1.26, 2.02)	0.65	0.01	2.77 (−0.49, 6.03)	0.10	−3.08 (−5.84, −0.33)	0.03	1.96 (−0.65, 4.57)	0.14
Total protein foods	2.39 (0.76, 4.03)	0.004	0.51	3.31 (0.14, 6.48)	0.04	1.10 (−1.65, 3.85)	0.43	2.97 (0.30, 5.64)	0.03
Seafood and plant proteins	−0.58 (−2.19, 1.03)	0.48	0.41	0.99 (−2.26, 4.24)	0.55	−0.24 (−3.08, 2.59)	0.87	−1.74 (−4.20, 0.72)	0.17
Fatty acids	−0.85 (−2.51, 0.80)	0.31	0.31	−3.05 (−6.49, 0.39)	0.08	0.32 (−2.37, 3.01)	0.82	−0.69 (−3.34, 1.96)	0.61
**HEI−2015 moderation components**									
Refined grains	−0.31 (−1.92, 1.30)	0.71	0.01	3.92 (0.55, 7.30)	0.02	−2.56 (−5.01, −0.10)	0.04	−0.30 (−3.08, 2.48)	0.83
Sodium	−1.40 (−3.11, 0.30)	0.11	0.15	1.15 (−1.93, 4.23)	0.47	−2.32 (−5.32, 0.67)	0.13	−2.71 (−5.52, 0.10)	0.06
Added sugar	3.94 (2.18, 5.69)	<0.0001	0.59	2.79 (−0.53, 6.12)	0.10	5.15 (2.05, 8.25)	0.001	3.76 (0.98, 6.53)	0.01
Saturated fats	−2.25 (−3.98, −0.52)	0.01	0.13	−2.27 (−5.52, 0.98)	0.17	−0.17 (−3.04, 2.69)	0.91	−4.42 (−7.36, −1.48)	0.003
**% energy from added sugar**	−3.10 (−4.53, −1.68)	<0.0001	0.27	−1.53 (−4.07, 1.02)	0.24	−4.44 (−6.94, −1.94)	0.001	−3.27 (−5.65, −0.89)	0.01

Note: Models were adjusted for age, sex, body mass index, self-reported race and ethnicity, education, smoking status, tea, coffee, alcohol, and total energy intake. The HEI-2015 includes 13 components that capture adequacy (i.e. total fruits, whole fruits, total vegetables, greens and beans, whole grains, dairy, total protein foods, seafood and plant proteins, and fatty acids) and moderation (i.e. refined grains, sodium, added sugars, and saturated fats) components. Most of the components are scored on a density basis (per 1,000 kcal or as a percentage of energy intake) where a higher score for adequacy components indicates greater consumption while a higher score for moderation components indicates lower consumption. The HEI-2015 scores are modeled as a 1 unit increase in the score. % energy from added sugar represents a change of 5% in energy from added sugar. *p* < 0.05 as described in the methods.

### HEI-2015 associations with genus-level presence and relative abundance

**Figure 2.**
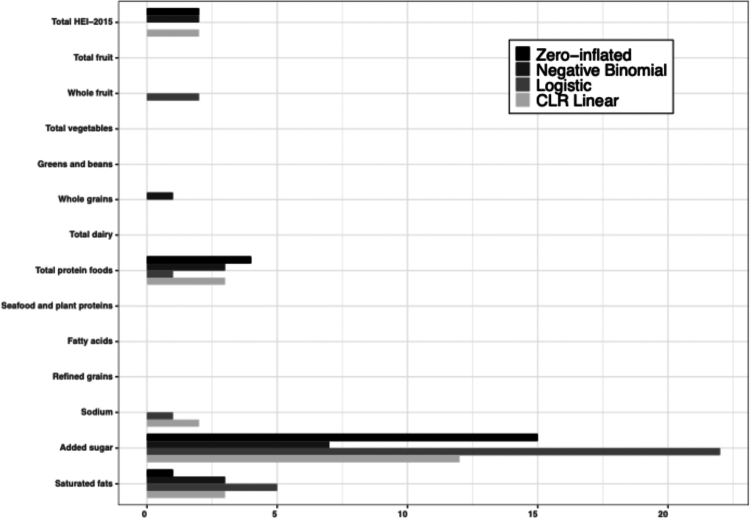
Number of genus-level associations (FDR *p*-value < 0.05) from the zero-inflated negative binomial, logistic, and CLR-transformed linear regression meta-analysis models for total HEI-2015 and HEI-2015 components. Zero-inflated negative binomial regression models were used for genera that had a weighted prevalence ranging from 5-95% and a weighted relative abundance greater than 0.1% (*N* = 21 genera); logistic regression models were used for genera with a weighted prevalence ranging from 5-95% but with a weighted relative abundance less than or equal to 0.1% (*N* = 60 genera); and CLR-transformed linear regression models were used for genera with a weighted prevalence greater than 95% (*N* = 20 genera).

At the genus-level, very few associations were observed with total HEI-2015, with significant genus-level associations (i.e. FDR *p*-value less than 0.05) detected for 2 genera (*Mogibacterium* and *Solobacterium*) for the zero-inflated component and 2 genera (*Bergeyella* and *Treponema 2*) for the negative binomial component of the zero-inflated negative binomial models, 0 genera for the logistic regression models, and 2 genera (*Oribacterium* and *Stomatobaculum*) for the CLR-transformed abundances in the linear regression models. For the HEI-2015 components, most of the associations were detected with the added sugar component, particularly for genus-level presence based on the zero-inflated term in the zero-inflated negative binomial model and the logistic regression model with 15 and 22 significant associations, respectively (Figure 2). For the zero-inflated negative binomial models, the strongest associations between added sugar and the zero-inflated term were for the associations with *Mogibacterium* (OR_5% energy from sugar_: 0.78; 95% CI: 0.70, 0.97), *Parvimonas* (OR_5% energy from sugar_: 0.79; 95% CI: 0.70, 0.89), [*Eubacterium*] *nodatum* group (OR_5% energy from sugar_: 0.80; 95% CI: 0.74, 0.88), and *Solobacterium* (OR_5% energy from sugar_: 0.81; 95% CI: 0.73, 0.91). Interestingly, most of the associations for higher added sugar consumption (i.e. lower HEI component score for added sugar) were inverse, such that there was a lower odds of detecting specific genera, including periodontal pathogens like *Porphyromonas*, *Peptostreptococcus*, and *Treponema 2* (**Supplemental Table 2**). For the negative binomial term from the zero-inflated negative binomial models, there were associations between multiple HEI components with the relative abundance of *Bergeyella*. Inverse associations with the relative abundance of *Bergeyella* were detected with the total HEI-2015 score (RR: 0.87; 95% CI: 0.82, 0.93), whole grains (RR 0.89; 95% CI: 0.83, 0.95), and saturated fats (RR: 0.85; 95% CI: 0.79, 0.92), while a positive association was seen with total protein foods (RR: 1.15; 95% CI: 1.08, 1.22). For added sugar, most of the associations with the negative binomial term were observed for genera with a statistically significant association with the zero-inflated term. For example, for a higher proportion of energy from added sugar, there was a lower odds of detecting *Porphyromonas* (OR: 0.85; 95% CI: 0.77, 0.92); and among individuals with detectable *Porphyromonas*, for a 5% higher proportion of energy from added sugar, the relative abundance of *Porphyromonas* was lower (RR: 0.92; 95% CI: 0.89, 0.96) (**Supplemental Table 2**).

For the logistic regression models with genera with a weighted prevalence of 5-95% and a weighted average relative abundance less than or equal to 0.1%, the HEI component for added sugar intake was associated with 22 out of the 60 genera tested. The strongest association was observed between added sugar and *Bifidobacterium* presence where a 5% greater proportion of energy intake from added sugar (i.e. lower HEI component score for added sugar) was associated with greater odds of detecting *Bifidobacterium* (OR: 1.22; 95% CI: 1.13, 1.31). Conversely, inverse associations were observed with the remaining statistically significant genera including presence of *Prevotella 2*, *Tannerella*, *Johnsonella*, and [*Eubacterium*] *yurii group* (**Supplemental Table 3**). For the linear regression models with CLR-transformed abundances of genera with a weighted prevalence greater than 95%, the proportion of energy from added sugar was positively associated with the relative abundances of 11 genera, including *Veillonella* (β: 0.06; 95% CI: 0.04, 0.09), *Neisseria* (β: −0.18; 95% CI: −0.27, −0.10), *Streptococcus* (β: 0.04; 95% CI: 0.03, 0.06), and *Fusobacterium* (β: −0.11; 95% CI: −0.16, −0.06) (**Supplemental Table 4**).

## Discussion

In this large cross-sectional analysis of three well-characterized US cohorts, we investigated the association between diet quality, measured by HEI-2015, and the oral microbiome. Our findings suggest that overall diet quality is generally not associated with the oral microbial community. However, the intake of added sugars, a key component of diet quality, was consistently associated with multiple measures of oral microbial diversity and with many specific bacterial genera across all three cohorts. Of all our measured dietary factors, added sugar intake was found to be the most strongly associated with the bacterial community overall (i.e. beta diversity). In addition, greater added sugar consumption was linked to lower alpha diversity and decreased odds of detecting genera such as *Porphyromonas*, *Parvimonas*, and *Tannerella*. Conversely, greater added sugar consumption was associated with greater odds of detecting *Bifidobacterium*. These results suggest that added sugar consumption is likely an important factor influencing the oral microbial communities of adults.

A previous study conducted within the Buffalo OsteoPerio cohort of the Women's Health Initiative (WHI) evaluated the association between the HEI-2020 (identical to HEI-2015) and the subgingival microbiome within 1,175 postmenopausal women. For alpha diversity, similar to our study, lower added sugar intake (i.e. an increase in the HEI score) was associated with greater alpha diversity. They also found a positive association between intake of total protein foods or seafood and plant proteins with alpha diversity. For the relative abundance of 31 selected taxa (3 phyla, 16 genera, and 12 species), the strongest associations were observed with overall HEI and the HEI components for total vegetables and seafood and plant proteins, but 6 taxa were significantly associated with added sugar intake including positive associations with decreased added sugar intake for the relative abundance of *Actinomyces species oral taxon 171*, *Bifidobacterium*, and *Spirochaetes* and inverse associations for *Firmicutes*, *Alloprevotella*, and *Ottowia*. [29] *Bifidobacterium* in our study was at a low relative abundance (< 0.1%), so we only tested the association with the presence of *Bifidobacterium*; however, unlike the previous WHI study, we found that lower added sugar intake was associated with lower odds of *Bifidobacterium* presence. Our results may differ from the WHI study due to the testing of different oral sites (i.e. subgingival sampling versus oral wash), population differences, or other methodological differences. Still, our overall findings are similar in that specific dietary components, but not overall diet quality, are associated with the oral microbiome.

Observed associations between added sugar consumption and the oral microbiome are plausible given previous studies considering the impact of sugar consumption on oral health. Sugar consumption has long been implicated in the development of caries [30] as have multiple specific oral bacteria [31]. Feeding studies of sugar have also demonstrated changes to the oral microbiome using both culture-based methods [32] and sequencing [33]. A systematic review of studies published between 2010 and June of 2021 identified eight studies of the relationship between sugar intake and the oral microbiome [34]. Similar to our findings, the majority of these studies found an association between higher sugar consumption and lower alpha diversity. In addition, differences in the presence or relative abundance of a number of bacterial genera and species were observed, with a number of studies identifying associations with *Streptococcus* [34]. A more recent study suggested that high sugar consumption may be affecting salivary pH mediated through changes to *Streptococcus*, *Neisseria*, *F0332*, *Veillonella*, and *Actinomyces* [35]. Additional research is needed to understand how added sugar intake impacts oral microbial communities and if the source (e.g. cakes, candy, soft drinks, fruit drinks) or type (e.g. white sugar, high-fructose corn syrup) of the added sugars results in differential effects on the microbiome as this could have important implications for dietary guidance.

There are several strengths of this study. The large sample size increased our power to detect modest associations. The inclusion of three distinct US cohorts and the evaluation of heterogeneity in the associations between the cohorts strengthens the validity of the observed associations across geographically diverse US adult populations. The use of similar, validated FFQs and consistent methodology for scoring the HEI-2015 allowed for a comprehensive assessment of diet quality and its components across three study populations. In addition, our microbiome analyses incorporated multiple diversity metrics and genus-level prevalence and abundance, using complementary statistical models that accounted for our complex sampling designs and potential confounders, including age, smoking, and BMI. This rigorous approach reduces bias and improves the robustness of our findings. However, several limitations warrant consideration. The cross-sectional design precludes establishing a temporal relationship between diet and the oral microbiome. In addition, measurement error is inherent in FFQs and may have resulted in misclassification of HEI-2015. The FFQ estimates habitual intake and therefore this study only estimates how an average reported diet may influence the oral microbiome and not an immediate effect of diet on the oral microbiome. Dietary intake was not always contemporaneous with the oral sample collection, particularly in the NIH-AARP where the FFQ was administered approximately 10 years before sample collection, but the consistency across cohorts with FFQs more proximate to the oral sample collection provides more confidence in these results. The oral microbiome was characterized using 16S rRNA gene sequencing of oral wash specimens. The use of 16S rRNA gene sequencing limits taxonomic resolution and functional inference, and the oral wash specimen may yield unique results compared to more targeted collections, such as subgingival plaque [36]. Future studies should incorporate shotgun metagenomic sequencing to further evaluate species-level and functional associations. Although we included data from three distinct US cohorts, the populations are not nationally representative and future studies should evaluate these associations within representative populations. Finally, despite adjusting for multiple confounders, residual confounding by unmeasured factors such as oral hygiene behaviors, oral conditions such as periodontal disease, or medication use like antibiotics cannot be excluded.

In conclusion, our study demonstrated that while overall diet quality was not strongly associated with the oral microbiome, added sugar consumption was consistently linked to oral microbial diversity and specific bacterial taxa in three US cohort studies. These findings highlight added sugars as a modifiable dietary factor that may influence oral microbial ecology. Future studies should evaluate whether the frequency or timing of added sugar consumption is influential to the composition of the oral microbiome in addition to whether added sugar intake interacts with other important determinants of oral microbiome composition such as smoking. Finally, longitudinal studies and mechanistic investigations are needed to clarify causal pathways and explore whether dietary interventions targeting sugar intake can beneficially modulate the oral microbiome to reduce risk of multiple adverse health conditions, including cancer.

## Supplementary Material

Supplemental_Figure.docx Supplemental_Figure.docx

Supplemental tables_20260123.xlsx Supplemental tables_20260123.xlsx

## Data Availability

Microbiome sequencing data is available at the Sequence Read Archive (SRA) under project number PRJNA801882 with limited metadata. For complete metadata, a data application will need to be approved from the Agricultural Health Study (www.aghealthstars.com), the NIH-AARP Diet and Health Study (www.nihaarpstars.com), and the Prostate, Lung, Colorectal, and Ovarian Cancer Screening Trial (www.cdas.cancer.gov).
