# Supplementary material for: Association between diet quality and the oral microbiome in three US cohort studies
Source: J Oral Microbiol. 2026 Feb 24;18(1):2635238. doi: 10.1080/20002297.2026.2635238 (PMC12934338; doi:10.1080/20002297.2026.2635238)
Supplement: Supplemental_Figure.docx [file ZJOM_A_2635238_SM5498.docx]

**Supplemental Figure 1.** Participant flow chart. For this study, data from the nested oral microbiome case-cohort study within the Agricultural Health Study, NIH-AARP Diet and Health Study, and the Prostate, Lung, Colorectal, and Ovarian Cancer Screening Trial was included. From that population, individuals who were missing demographic, lifestyle, dietary, or microbiome data, had prevalent cancer at oral wash collection, and those with extreme caloric intake (<600 kcal or >5000 kcal) were excluded.


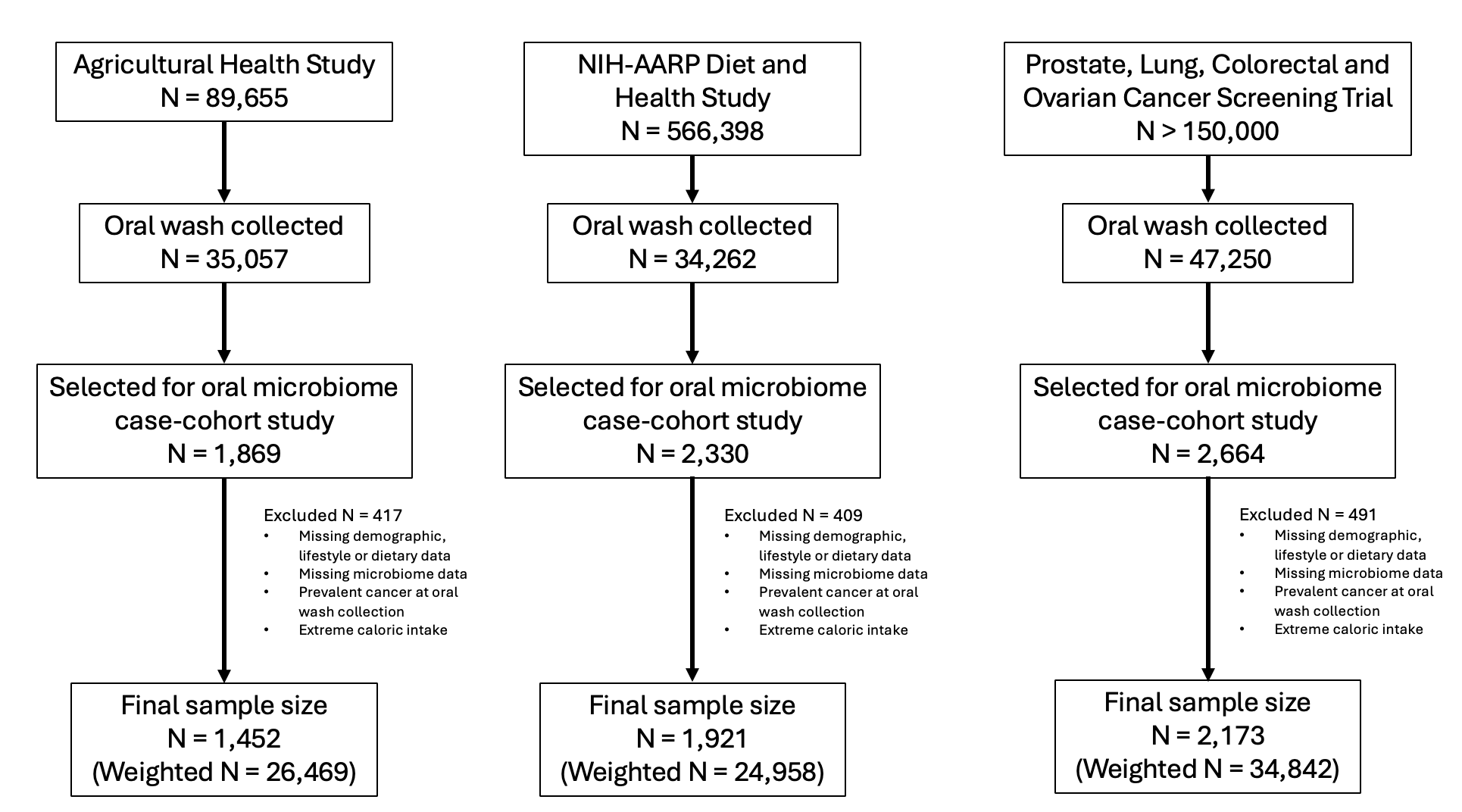


**Supplemental Figure 2.** Overall variability in the beta-diversity matrices (i.e., Bray-Curtis, unweighted UniFrac, and weighted UniFrac) explained by the confounders only (i.e., demographic, lifestyle, and other dietary factors), the total Healthy Eating Index-2015 (HEI) and HEI-2015 components, and all variables estimated using the FastAdonis function. The diversity matrices were pooled across the three cohorts and each panel represents the variance explained by multivariable regression models accounting for simultaneous adjustment of relevant variables.

*
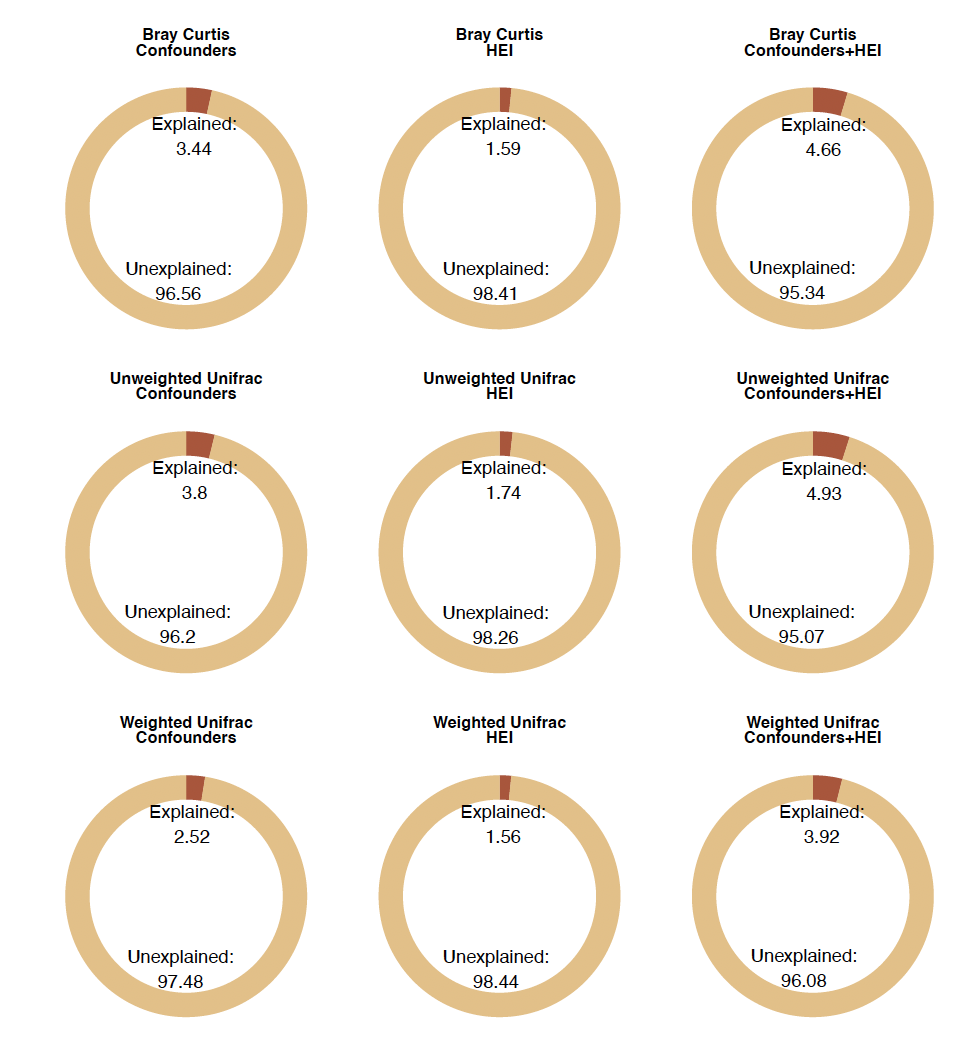
*
